# Study of Antioxidant Activity of Some Medicinal Plants Having High Content of Caffeic Acid Derivatives

**DOI:** 10.3390/antiox9050412

**Published:** 2020-05-12

**Authors:** Agnieszka Tajner-Czopek, Mateusz Gertchen, Elżbieta Rytel, Agnieszka Kita, Alicja Z. Kucharska, Anna Sokół-Łętowska

**Affiliations:** 1 Department of Food Storage and Technology, Faculty of the Biotechnology and Food Science, Wrocław University of Environmental and Life Sciences, Chełmońskiego St. 37, 51-630 Wrocław, Poland; mateusz.gertchen@upwr.edu.pl (M.G.); elzbieta.rytel@upwr.edu.pl (E.R.); agnieszka.kita@upwr.edu.pl (A.K.); 2 Department of Fruit, Vegetable and Plant Nutraceutical Technology, Faculty of the Biotechnology and Food Science, Wrocław University of Environmental and Life Sciences, Chełmońskiego St. 37, 51-630 Wrocław, Poland; alicja.kucharska@upwr.edu.pl (A.Z.K.); anna.sokol-letowska@upwr.edu.pl (A.S.-Ł.)

**Keywords:** phenolic acids, caffeic acid derivatives, medicinal plants, extraction, antioxidant activity, ABTS, DPPH

## Abstract

Recently, there has been increasing interest in medicinal plants, due to their content of health-promoting compounds, e.g., caffeic acids derivatives. Hence, the aim of this work was to study the antioxidant activity of extracts obtained from the following medicinal plants: caraway (*Carum carvi* L.), coltsfoot (*Tussilago farfara* L.), dandelion (*Taraxacum officinale* F.H.Wigg.), lovage (*Levisticum officinale* L.), tarragon (*Artemisia dracunculus* L.) and white mulberry (*Morus alba* L.), characterized by their high content of caffeic acid derivatives. The water-ethanolic extracts were characterized on average by about 9 times higher contents of caffeic acid derivatives level than water extracts. Both in water and water-ethanolic extracts, the dominant phenolic acid was 5-CQA (5-*O*-caffeoylquinic acid) and 3,4-diCQA (3,4-dicaffeoylquinic acid), then CCA-1 (chicoric acid isomer 1), which appeared only in water-ethanolic extracts. Extracts from dandelion contained compounds such as CTA (caftaric acid), CCA-1 (chicoric acid isomer 1) and CCA-2 (chicoric acid isomer 2), which were not detected in other plant extracts examined in this work. The water-ethanolic extracts from coltsfoot and tarragon were characterized by a high content of di-caffeoylquinic acids, especially 3,4-diCQA and 3,5-diCQA, respectively. It has been stated that there is a positive correlation between caffeic acid derivatives and antioxidant activity (radical cation scavenging activity (ABTS) and radical scavenging activity (DPPH)), especially in water-ethanolic extract of medicinal plants.

## 1. Introduction

In recent times, interest in the application of plant extracts in the food industry has continued to grow, mainly because of their antioxidant properties and associated health-promoting effects for humans. These properties of extracts are connected to the presence of vitamins, minerals and different phenolic compounds in plants [1,2,3]. In the group of phenolic compounds, special attention should be paid to phenolic acids, which can counteract the development of coronary heart disease, inflammation, diabetes and cancer [3,4,5,6].

In plant tissues, there are a number of phenolic acids, including chlorogenic acids (CGA). Chlorogenic acids are a family of esters formed between certain phenolic acids (*trans-*cinnamic acids) and quinic acid. The chlorogenic acids group includes: caffeoylquinic acids (CQAs), dicaffeoylquinic acids (di-CQA) and other caffeic acid derivatives [7,8]. The group of CQAs includes the following acids: 5-*O*-caffeoylquinic acid (5-CQA), 4-*O-*caffeoylquinic acid (4-CQA) and 3-*O*-caffeoylquinic acid (3-CQA) [8,9]. Benefits for human health associated with the increased consumption of CQAs include antiviral, hepatoprotective and hypoglycaemic effects [3,6,10].

Caffeoylquinic acids are found in almost every existing plant, but the major dietary source for humans is coffee [8,9,10,11]. There is also a relatively high content of these components in potatoes (*Solanum tuberosum*), especially in coloured-flesh varieties [12,13]. Rytel et al. [12] reported that the content of phenolic acids in purple-fleshed potatoes is more than 2-fold higher than that in traditional, yellow-fleshed potatoes, whereas Silveira et al. [14] stated that the quantities of these compounds are 3–4 times higher in coloured potato tubers.

Well-known herbs such as oregano, rosemary and thyme are good sources of phenolic compounds. Wojdyło et al. [15] confirmed the high content of caffeic (649 mg·100 g^−1^ dry matter(d.m.)) and 3-*O*-caffeoylquinic acid (96 mg·100 g^−1^ d.m.) in oregano. Rosemary and thyme are known for their high amounts of caffeic acid and ferulic acid [15]. These medicinal plants are popular among consumers and food producers, but there are different plants, which are both better and less well-known, whose extracts can be used as additives for food, because of their high content of different pro-health compounds.

One of these plants is caraway (*Carum carvi* L.), which is used in folk medicine for the treatment of diarrhoea and bronchopulmonary disorders, or to improve liver function. The major components of caraway are carvacrol, carvone, α-pinene, limonene, γ-terpinene, linalool, carvenone and *p*-cymene [16], as well as various phenolic acids, such as gallic, syringic, neochlorogenic, cryptochlorogenic and caffeic acid [17].

A well-known herb in Chinese medicine and Eastern Europe belonging to the *Asteraceae* family is coltsfoot (*Tussilago farfara* L.). The antioxidant effect of this plant is due to, among other things, quercetin-glycosides [18]. Dandelion (*Taraxacum officinale* F.H. Wigg.) is a good source of minerals, especially calcium. This plant is used as a therapeutic treatment due to its choleretic, diuretic, anti-rheumatic and anti-inflammatory properties. Cinnamic acid, coumarins and flavonoids are among the phytochemical compounds isolated from dandelion [19,20]. Another plant with strong antioxidant properties offering health benefits is lovage (*Levisticum officinale* L.). It is an herbaceous plant with a strong celery-like aroma, originally native to Southwest Asia and Southern Europe. Both the roots and leaves of lovage contain phenolic compounds [21].

Another plant that exhibits medicinal properties is tarragon (*Artemisia dracunculus* L.). Tarragon as a medicinal plant is used for the treatment of stomach pains, pyrexia, diabetes and parasitic or bacterial infections [22]. Alcoholic extracts from this plant can inhibit platelet aggregation. Major constituents of the plant are estragole, phellandrene, iodine, tannins, methyl coumarins, chavicol and rutin [23,24]. Also, the white mulberry (*Morus alba* L.) can be a source of antioxidant compounds. The leaf extracts from white mulberry exhibit, for example, anti-diabetic properties [25].

Herbs are readily available; their consumption along with food is constantly increasing, which means that the amount of valuable phenolic compounds delivered to humans is also increasing. They are consumed in the fresh form, after drying or in the form of plant extracts. Herbs and spices traditionally used in food preparation provide attractive sensory features, i.e., taste, smell and colour [26,27]. In contrast, new possibilities of using plant extracts are associated with the possibility of, for example, reduction of fat and acrylamide in the ready products. Urbančič et al. [28] reported that the use of rosemary extract as an oil additive not only reduced the acrylamide and oil content of fried potatoes but also stabilized the heated fat and prevented it from rancidity. Morales et al. [29] showed that the addition of the extract from green tea, cinnamon and oregano to immerse the potato strips had an effect on acrylamide reduction in fries by about 62%, 39% and 17% respectively, compared to the control sample. The phenolic compounds contained in medicinal plants can reduce the amount of acrylamide (AA) formation in the finished product by partially blocking the formation of intermediate Maillard reaction products and thus reducing the amount of toxic compound [30].

Extracts of the above-mentioned medicinal plants could be used in the food industry to improve the quality and healthiness of products. The cheapest way to obtain plant extracts is preparation with water to extraction; although, better results are observed when using ethanol or methanol, however methanol, because of toxicity, cannot be used in food production.

Despite extensive research into the chemical composition of various plants, detailed information on the content of caffeic acid derivatives in herb and spice plant extracts is not collected in the scientific literature. Therefore, the authors undertook the task of thoroughly analysing a number of these better and less well-known plants in terms of the content of phenolic acids that are valuable for the human body and determining their amount depending on the extractant used in the work.

The aim of the work was to study the antioxidant activity of extracts obtained from the following medicinal plants: caraway (*Carum carvi* L.), coltsfoot (*Tussilago farfara* L.), dandelion (*Taraxacum officinale* F.H.Wigg.), lovage (*Levisticum officinale* L.), tarragon (*Artemisia dracunculus* L.) and white mulberry (*Morus alba* L.), characterized by their high content of caffeic acid derivatives.

## 2. Materials and Methods

### 2.1. Material

The material comprised dried seeds of caraway (*Carum carvi* L.), as well as leaves of coltsfoot (*Tussilago farfara* L.), dandelion (*Taraxacum officinale* L.), lovage (*Levisticum officinale* L.), tarragon (*Artemisia dracunculus* L.) and white mulberry (*Morus alba* L.), and were purchased from a local producer and grower situated in the Lower Silesia. The plants came from the 2018 growing season. In order to prepare extracts, raw plant material was freeze-dried in lyophilizer (type 5411, BOC Ltd., Edwards, England) and crushed in an electric mill (FA-5485 SP-742, TZS First, Austria). Ground dry material was packed in plastic closable packaging, after which the samples were used directly for analyses (at room temperature).

### 2.2. Chemicals

Phenolic acid standards, i.e., 5-*O*-caffeoylquinic acid (5-CQA), 4-*O*-caffeoylquinic acid (4-CQA) as well as 3-*O*-caffeoylquinic acid (3-CQA) were purchased from TRANS MIT GmbH (Giessen, Germany). Caftaric acid was bought from Cayman Chemical Co. (Ann Arbor, MI, USA). Dicaffeoylquinic acid, *p*-coumaric acid and ferulic acid were purchased from Extrasynthese (Genay, France), whereas Folin-Ciocalteu reagent, sodium hydroxide,6-hydroxy-2,5,7,8-tetramethylchroman-2-carboxylic acid (Trolox), 2,2-azino-bis-3-ethylbenzothiazoline-6-sulphonic acid diammonium salt (ABTS), 1,1-diphenyl-2-picrylhydrazyl radical DPPH, sodium hydrogen sulfite (NaHSO_3_) and formic acid were purchased from Sigma-Aldrich Chemical Co. (Steinheim, Germany). Acetonitrile was bought from POCh (Gliwice, Poland). All reagents were of analytical grade.

### 2.3. Extraction

**Figure 1:**
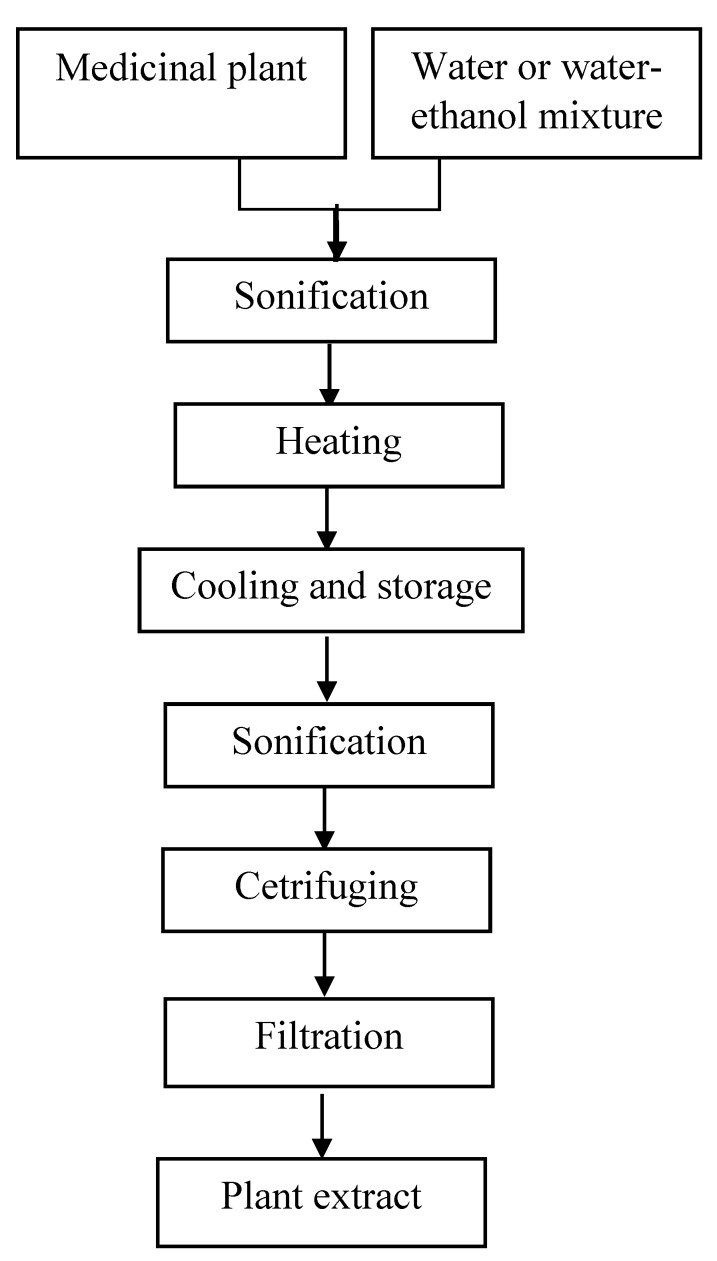
Scheme of water and water-ethanolic extract preparation.

Extraction was carried out using water and 50% (*v*/*v*) ethanol in water. Extracts from medicinal plants were prepared according to the method of Bucić-Kojić et al. [31], with some modifications. The extracts were prepared as follows: 2 grams of ground plant material were mixed with 100 mL of distilled water (water extracts) or 100 mL of a mixture (1:1) of distilled water and ethyl alcohol (water-ethanolic extracts) with the addition of 0.1% NaHSO_3_. The obtained mixture was sonicated in an ultrasonic shaker (UM-2, Unitra-Unima Olsztyn, Poland) for 15 min and heated at 80 °C for 5 min. Afterwards, the mixture was cooled down to room temperature and stored at 4 °C for 12 h. Next, the mixtures were re-sonicated and centrifuged at 5000 rpm (MPW-351R, Mpw Med. Instruments, Poland) for 10 min (Figure 1). Finally, extracts were used for analyses of phenolic compounds and antioxidant activity (AA).

### 2.4. HPLC Analysis of Caffeic Acid Derivatives

The concentrations of caffeic acid derivatives, e.g., caffeoylquinic acids (3-*O*-caffeoylquinic acid, 4-*O*-caffeoylquinic acid and 5-*O*-caffeoylquinic acid), dicaffeoylquinic acids (3,4-diCQA—3,4-dicaffeoylquinic acid; 3,5-diCQA—3,5-dicaffeoylquinic acid; 4,5-diCQA—4,5-dicaffeoylquinic acid), and other compounds (CTA—caftaric acid; CCA-1—chicoric acid isomer 1; CCA-2—chicoric acid isomer 2) were performed and calculated on the basis of the HPLC assay described by Kucharska et al. [32] with some modifications. The Dionex Ultimate 3000 HPLC system (Germering, Germany), with a PDA detector, and Cadenza Imtakt column CD–C18 (75 × 4.6 mm, 5 μm) with a guard column, was used. Reagents: A—4.5% formic acid, B—acetonitrile, flow rate 1 mL/min, gradient: 0–1 min 5% B, 20 min 25% B, 21 min 100% B, 26 min 100% B, 27 min 5% B. The column was operated at 30 °C and compounds were detected at 320 nm. Caffeic acid derivatives were expressed as mg of 5-*O*-caffeoylquinic acid equivalents ·g^−1^ of medicinal plant.

### 2.5. Antioxidant Activity (AA): Free-Radical-Scavenging Ability by the Use of an ABTS Radical Cation

The antioxidative activity of medicinal plant extracts was determined using the ABTS assay according to Re et al. [33]. 0.06 mL samples of extract were mixed with 3 mL of ABTS solution with measured absorption of 0.700 at a wavelength of 734 nm. After 6 min, the absorbance of samples was measured using a Rayleigh UV-2601 spectrophotometer (BRAIC, China). Each sample was tested in triplicate. The data were expressed as µmol Trolox equivalent (TE)·g^−1^ d.m.

### 2.6. Antioxidant Activity (AA): Free-Radical-Scavenging Ability by the Use of a DPPH Radical

The antiradical activity of medicinal plant extracts was determined using a DPPH assay according to Yen and Chen [34]. 0.2 mL samples of extract and 0.3 mL of distilled water were mixed with 2 mL of 0.05 mM DPPH ethanolic solution. After 10 min of incubation at room temperature in the dark, the absorbance was measured with a Rayleigh UV-2601 spectrophotometer (Beijing, China) at 517 nm. All determinations were performed in triplicate. Results were expressed as µmol Trolox equivalent (TE)·g^−1^ d.m.

### 2.7. Statistical Analysis

The obtained study results were subjected to statistical analysis using Statistica v.13 software. The experimental data were processed by one-way analysis of variance (ANOVA). Homogeneous groups were determined using Duncan’s test (at a significance level of *p* ≤ 0.05 and *p* ≤ 0.01). The correlation analysis was done to determine the strength and nature of the link between variables [35]. All determinations were carried out in three technological replicates, and the results represent mean values.

## 3. Results and Discussion

**Figure 2:**
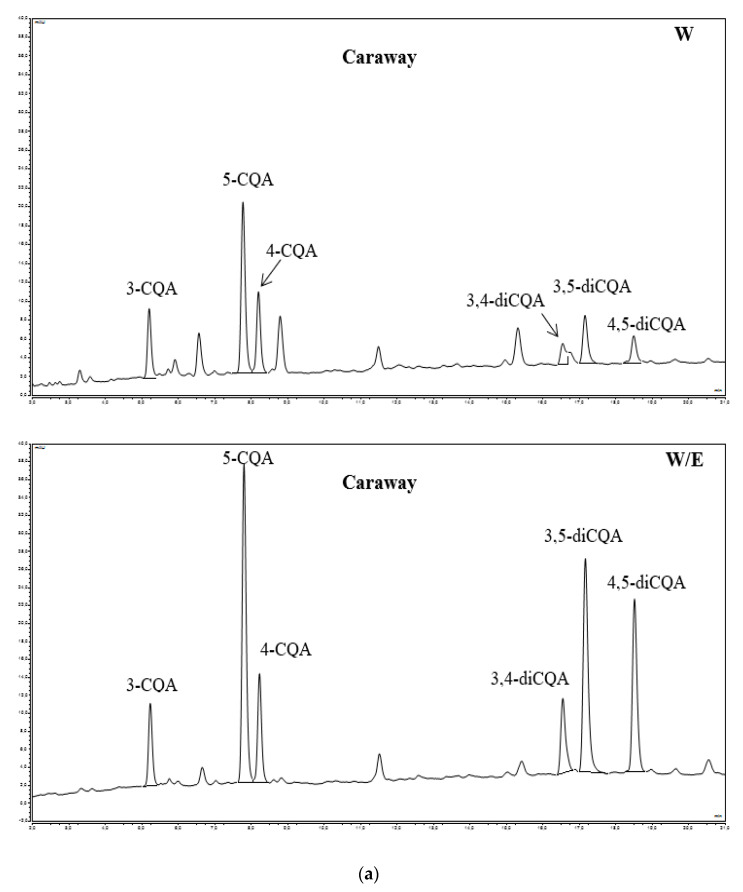

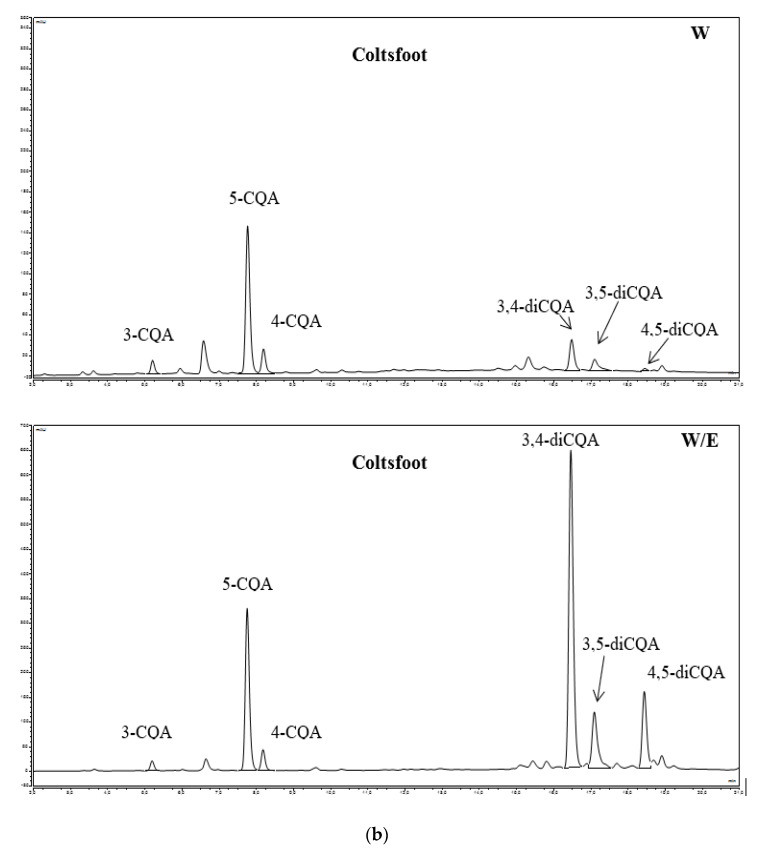

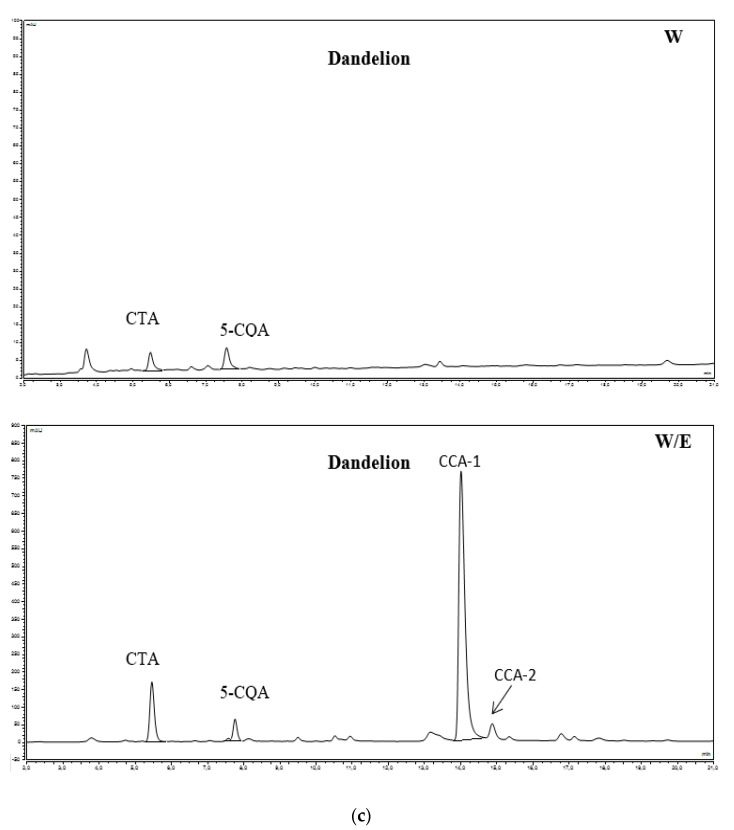

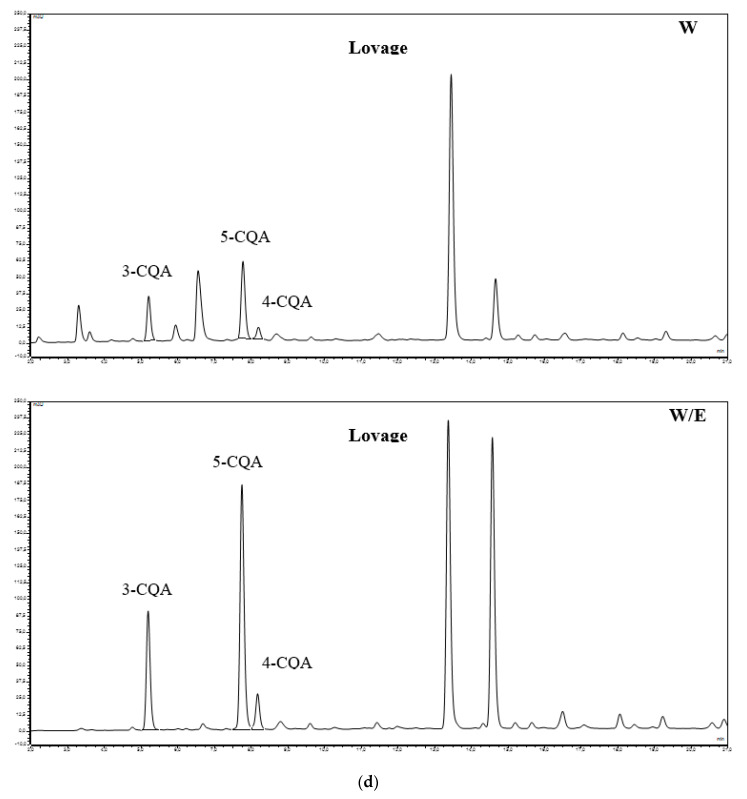

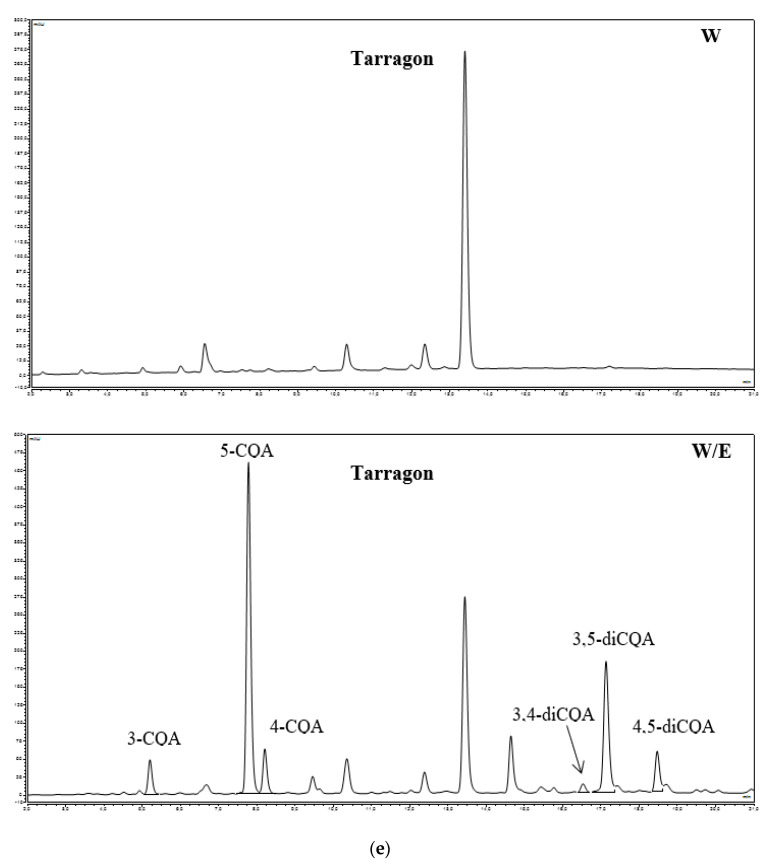

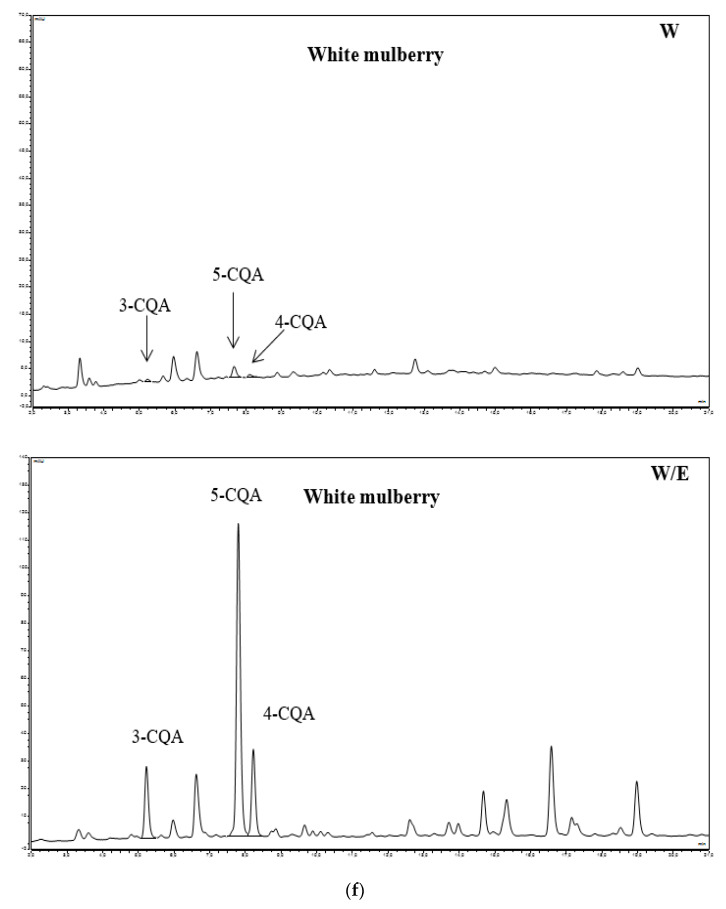
(**a**) HPLC-PDA chromatograms (320 nm) of phenolic acids of caraway water extracts (W) and water-ethanolic extracts (W/E); 3-CQA—(3-O-caffeoylquinic acid; neochlorogenic acid); 5-CQA—(5-O-caffeoylquinic acid; chlorogenic acid); 4-CQA—(4-O-caffeoylquinic acid; cryptochlorogenic acid); 3,4-diCQA—(3,4-dicaffeoylquinic acid); 3,5-diCQA—(3,5-dicaffeoylquinic acid); 4,5-diCQA—(4,5-dicaffeoylquinic acid). (**b**) HPLC-PDA chromatograms (320 nm) of phenolic acids of coltsfoot water extracts (W) and water-ethanolic extracts (W/E); 3-CQA—(3-O-caffeoylquinic acid; neochlorogenic acid); 5-CQA – (5-O-caffeoylquinic acid; chlorogenic acid); 4-CQA—(4-O-caffeoylquinic acid; cryptochlorogenic acid); 3,4-diCQA—(3,4-dicaffeoylquinic acid); 3,5-diCQA—(3,5-dicaffeoylquinic acid); 4,5-diCQA—(4,5-dicaffeoylquinic acid). (**c**) HPLC-PDA chromatograms (320 nm) of phenolic acids of dandelion water extracts (W) and water-ethanolic extracts (W/E); CTA—(caftaric acid); 5-CQA—(5-O-caffeoylquinic acid; chlorogenic acid); CCA-1—(chicoric acid isomer 1); CCA-2—(chicoric acid isomer 2). (**d**) HPLC-PDA chromatograms (320 nm) of phenolic acids of lovage water extracts (W) and water-ethanolic extracts (W/E); 3-CQA—(3-O-caffeoylquinic acid; neochlorogenic acid); 5-CQA—(5-O-caffeoylquinic acid; chlorogenic acid); 4-CQA—(4-O-caffeoylquinic acid; cryptochlorogenic acid). (**e**) HPLC-PDA chromatograms (320 nm) of phenolic acids of tarragon water extracts (W) and water-ethanolic extracts (W/E); 3-CQA—(3-O-caffeoylquinic acid; neochlorogenic acid); 5-CQA—(5-O-caffeoylquinic acid; chlorogenic acid); 4-CQA—(4-O-caffeoylquinic acid; cryptochlorogenic acid); 3,4-diCQA—(3,4-dicaffeoylquinic acid); 3,5-diCQA—(3,5-dicaffeoylquinic acid); 4,5-diCQA—(4,5-dicaffeoylquinic acid). (**f**) HPLC-PDA chromatograms (320 nm) of phenolic acids of white mulberry water extracts (W) and water-ethanolic extracts (W/E); 3-CQA—(3-O-caffeoylquinic acid; neochlorogenic acid); 5-CQA—(5-O-caffeoylquinic acid; chlorogenic acid); 4-CQA—(4-O-caffeoylquinic acid; cryptochlorogenic acid).

**Figure 3:**
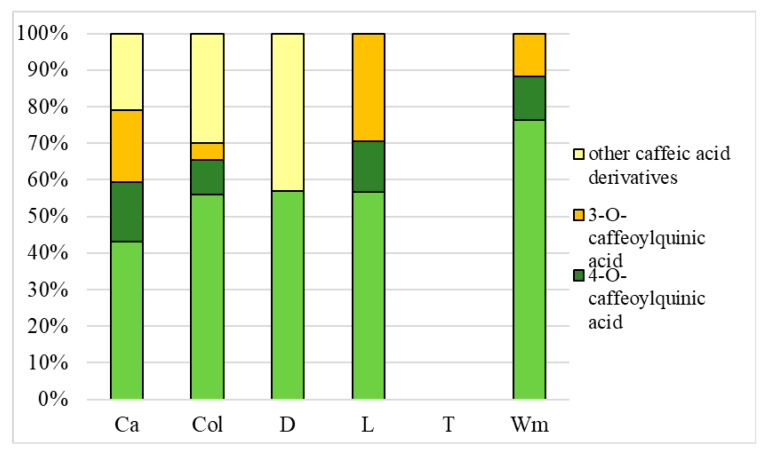
Percentage concentration of caffeic acid derivatives in water extracts. Ca—caraway; Col—Coltsfoot; D—dandelion; L—lovage; T—tarragon; Wm—white mulberry; 5-CQA—(5-*O*-caffeoylquinic acid; chlorogenic acid); 4-CQA—(4-*O*-caffeoylquinic acid; cryptochlorogenic acid); 3-CQA—(3-*O*-caffeoylquinic acid; neochlorogenic acid).

**Figure 4:**
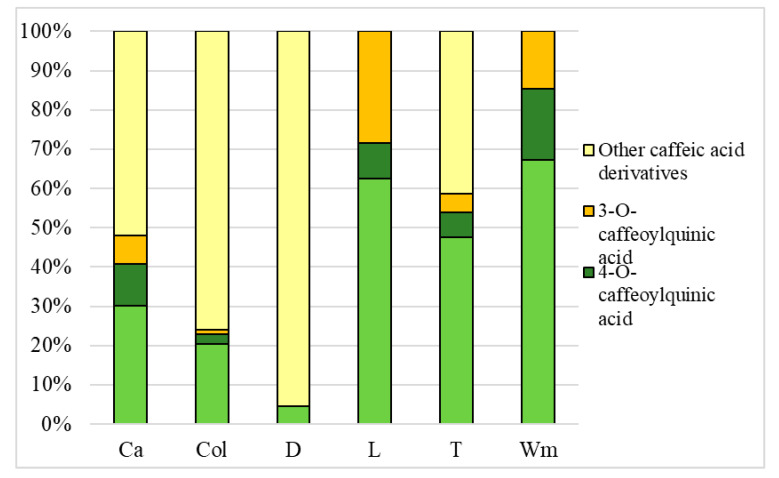
Percentage concentration of caffeic acid derivatives in water-ethanolic extracts. Ca—caraway; Col—Coltsfoot; D—dandelion; L—lovage; T—tarragon; Wm—white mulberry; 5-CQA—(5-*O*-caffeoylquinic acid; chlorogenic acid); 4-CQA—(4-*O*-caffeoylquinic acid; cryptochlorogenic acid); 3-CQA—(3-*O*-caffeoylquinic acid; neochlorogenic acid).

The chromatograms of caffeic acid derivatives contained in medicinal plants extracts, measured by HPLC at 320 nm, are shown in Figure 2a–f. The predominant caffeic acid and their derivatives in all investigated medicinal plants extracts are displayed in Table 1, and Figure 3 and Figure 4, respectively.

The investigated compounds were identified by their HPLC retention times, elution order, spectra of the individual peaks (UV/Vis), spectral data and by comparison with scientific literature. According to the retention time and elution order, the peaks in these chromatograms (Figure 2a–f) were identified as caffeoylquinic acids: 3-CQA (3-*O*-caffeoylquinic acid), 4-CQA (4-*O*-caffeoylquinic acid) and 5-CQA (5-*O*-caffeoylquinic acid), dicaffeoylquinic acids: 3,4-diCQA (3,4-dicaffeoylquinic acid), 3,5-diCQA (3,5-dicaffeoylquinic acid), 4,5-diCQA (4,5-dicaffeoylquinic acid), and other caffeic acid compounds: CTA (caftaric acid), CCA-1 (chicoric acid isomer 1) and CCA-2 (chicoric acid isomer 2), which is in agreement with the results of Craig et al. [8] and Clifford et al. [36]. These compounds exhibited UV/Vis absorption maxima (λ_max_) at 325 nm (3-CQA), 326 nm (5-CQA, 4-CQA, 3,4-diCQA), 327 nm (3,5-diCQA, 4,5- diCQA), 328 nm (CTA, CCA-1) and 329 nm (CCA-2). It was found that caffeoyloquinic acids (3-CQA, 5-CQA, 4-CQA) and dicaffeoylquinic acids (3,4-diCQA, 3,5-diCQA, 4,5-diCQA) were present in aqueous and aqueous-ethanolic extract of caraway and coltsfoot and in aqueous-ethanolic extract of tarragon, however 3-CQA, 5-CQA and 4-CQA content was found in aqueous and aqueous-ethanolic extract of lovage and white mulberry. The presence of CCA-1 (chicoric acid isomer 1) and CCA-2 (chicoric acid isomer 2) was found only in the aqueous-ethanol extract of dandelion, but a small amount of CTA and 5-CQA was found in both extracts from this plant. Whereas, none of investigated compounds were found in the aqueous extract of tarragon (Figure 2a–f; Table 1).

There is a lack of detailed information in the literature about quantitative analysis of other caffeic acids (especially of caffeic acid derivatives), so information described below are valuable in works on medicinal plants. It was reported that plant extracts were characterised by various contents of caffeic acid derivatives depending on the types of plant and solvent used (Table 1).

Among water extracts, coltsfoot extract contained the highest total amount of the analysed compounds (16.18 mg CQA·g^−1^), whereas very small amounts were noted in total white mulberry extract (0.18 mg CQA·g^−1^). Water-ethanolic extracts were characterised by higher amounts of caffeic acids compounds than water extracts (on average by about 9 times). The highest content of the total investigated compounds was found in the coltsfoot water-ethanolic extract (101.15 mg CQA·g^−1^) and then in the dandelion water-ethanolic extract (94.93 mg CQA·g^−1^). In the tarragon water-ethanolic extract, the amount of analysed compounds was also high and equal to 60.81 mg CQA·g^−1^, while caffeic acid derivatives were not found in the water extract of tarragon. The lowest content of total analysed acids was found in water extract of white mulberry (0.18 mg CQA·g^−1^).

It has been found that among all other caffeic acid derivatives, the highest quantity was of CCA-1 acid in water-ethanolic extract of dandelion. However, generally, both in water and water-ethanolic extracts, the dominant phenolic acid was 5-CQA (5-*O*-caffeoylquinic acid). The content of that compound in water extracts ranged from 0.14 mg CQA·g^−1^ (white mulberry) to 9.41 mg CQA·g^−1^ (coltsfoot), while from 2.23 mg CQA·g^−1^ (caraway) to 28.92 mg CQA·g^−1^ (tarragon) in water-ethanolic extracts. 5-*O*-caffeoylquinic acid represented 40% to 73% of all of the extracted caffeic acid derivatives. It was stated that both extracts were characterised by the significantly smaller amounts of 3-*O*-caffeoylquinic acid and 4-*O*-caffeoylquinic acid among the group of caffeyolqunic acids (average about 7-times lower than compared to the amount of acid 5-CQA). It has been stated that the highest content of 3-CQA (3-*O*-caffeoylquinic) was in water-ethanolic extract of tarragon (3.86 mg CQA·g^−1^) and 4-CQA (4-*O*-caffeoylquinic) in water-ethanolic extract of coltsfoot (2.53 mg CQA·g^−1^). However, the content of both compounds was not found in water and water-ethanolic extract of dandelion and water-ethanolic extract of tarragon.

It has been found that the water-ethanolic extract from coltsfoot and tarragon was characterised by a high content of di-caffeoylquinic acids, especially 3,4-diCQA and 3,5-diCQA (51.58 mg CQA·g^−1^ and 18.58 mg CQA·g^−1^, respectively).

Extracts from dandelion contained compounds such as CTA (caftaric acid), CCA-1 (chicoric acid isomer 1) and CCA-2 (chicoric acid isomer 2), which were not detected in other plant extracts examined in this work. It was stated that the highest content of CCA-1 (chicoric acid isomer 1) was found in water-ethanol of dandelion (73.83 mg CQA·g^−1^). The water-ethanolic extract of this plant also contained CTA (11.97 mg CQA·g^−1^) and CCA-2 (4.73 mg CQA·g^−1^), while in the water extract, there was a small amount of CTA (0.39 mg CQA·g^−1^). Generally, it was stated that among the water extracts, the highest content of the total studied compounds was characterised by coltsfoot.

Investigation of phenolic compound contents in coltsfoot was carried out by Dobravalskytė et al. [37]. They conducted analyses of coltsfoot extracts and detected higher caffeoylquinic acid using the HPLC-UV method. Research conducted by this author confirmed the occurrence of phenolic acid in coltsfoot extracts, as well as other phenolic acids such as dicaffeoylquinic and quinic acid. Ivanov [38] found phenolic (e.g., chlorogenic acid and chicoric acid) in dandelion extracts; depending on the extraction method, the content of that compound ranged from 18 mg·100 g^−1^ d.m. to 37 mg·100 g^−1^ d.m and from 484 mg·100 g^−1^ d.m. to 3148 mg·100 g^−1^ d.m., respectively. It is noteworthy that better extraction results were obtained by using a 50% water-ethanolic mixture as the extractant than 95% ethanol, while part of the investigated compounds were not detected in water extracts. Tsai et al. [39] stated that 50% ethanol is most favourable for extracting individual caffeic acid derivatives from plant *Echinacea purpurea*. Mirjalili et al. [40] analysed extracts from the dried leaves of lovage, and confirmed that caffeoyoloquinic acids are the main phenolic acids in those extracts. The authors stated that the content of these compounds in dried lovage leaves was 2–60-fold lower in comparison to caffeoylquinic acids.

Lin and Harnly [41] confirmed, by using the LC-PDA-ESI/MS method, that tarragon contained caffeoylquinic acids (3-CQA, 4-CQA and 5-CQA). On the other hand, Khezrilu et al. [42] did not find any derivatives of those acids in tarragon extract, but they identified other phenolic compounds, i.e., gallic acid, p-hydroxy benzoic acid, vanillic acid, p-coumaric acid, syringic acid, ferulic acid and sinapic acid. However, it should be noted that the authors used methanol as a solvent in their research. Chu et al. [43] reported that phenolic acids in white mulberry included caffeic acid, gallic acid and chlorogenic acid. Memon et al. [44] reported that caffeoyoloquinic acids were the dominant acids in white mulberry leaf extracts and the amount of these compounds varied from 47.9 mg·100 g^−1^ to 64.73 mg·100 g^−1^ depending on extraction method. In the mentioned works, none used the water and water-ethanol methods to extract compounds from the six selected medicinal plants investigated in this research.

Based on the results presented in Figure 3 and Figure 4, differences in the percentage concentration of all caffeic acid derivatives can be observed. In water extracts of caraway, coltsfoot and dandelion, caffeyolquinic acids represented 68% of the extracted caffeic acid derivatives.

In contrast, in water-ethanolic extracts of these plants, the content of other caffeic acid derivatives was dominant and represented about 78% of all extracted compounds. The content of other caffeic acid derivatives was not found in both the water and water-ethanolic extract from lovage and white mulberry, and the share of caffeyolquinic acids in both types of extracts was comparable. Differences between these two types of eluents can also be observed in tarragon. Caffeic acid derivatives (caffeyolquinic acids and other caffeic acid derivatives) were not detected in water extracts of tarragon, yet they represented about 58% and 42% of all phenolic compounds in water-ethanolic extract, respectively. It was stated that regardless of the eluent used, the dominant constituent in most of the analysed samples was 5-CQA (5-*O*-caffeoylquinic acid). Turkmen et al. [45], who examined the influence of extraction solvents on polyphenol concentrations, reported that 50% ethanol solvent is about 2.5-fold more effective than water. Also, Tan et al. [46] confirmed that the water-ethanol mixture is better for polyphenol extraction than distilled water, although they used a 60% ethanol-water mixture. Pudziuvelyte et al. [47] stated that the amount of compounds extracted from the plant is influenced by the type of solvent used to prepare the extract, the extraction process parameters and the anatomical parts of the plants used.

It was stated that the highest antioxidant activity in the analysed water extracts assayed with the ABTS method was determined in the coltsfoot extract (ca. 321 µmol TE·g^−1^ d.m.), whereas the lowest activity was detected in white mulberry extract (ca. 59 µmol TE·g^−1^ d.m.) (Table 2).

The antioxidant activity (AA) of the water-ethanolic extracts from the investigated plants obtained by the ABTS method was about 1.6-fold higher on average compared to extracts obtained using water as an extractant. The biggest difference was observed in white mulberry extracts, where antioxidant activity was 3-fold higher. With regard to the content of caffeic acids derivatives, the highest AA among water-ethanolic extracts was also observed in tarragon extract (ca. 406 µmol TE·g^−1^ d.m.), which was characterised by the highest amount of caffeyolqunic acids (3-CQA, 4-CQA and 5-CQA) and dicaffeyolqunic acid (3,5-diCQA) (Table 1). Both water and water-ethanolic extracts of coltsfoot were characterised by the highest AA measured by the DPPH method (ca. 52 µmol TE·g^−1^ d.m. and ca. 269 µmol TE·g^−1^ d.m., respectively) (Table 2), which could be related to the fact that coltsfoot was characterised by the highest amount of dicaffeyolqunic acid (3,4-diCQA and 4,5-diCQA) and higher amounts of 3,5-diCQA and 5-CQA in water-ethanolic extract, as well as the highest content of these compounds in water extract (Table 1). Similar to the ABTS assay results, the lowest DPPH antioxidant activity among water-ethanolic extracts was determined in extracts from white mulberry, which also had the lowest AA among water extracts, probably because the white mulberry contained only a small amount of caffeyolqiunic acids (3-CQA, 4-CQA and 5-CQA) both in water and water-ethanolic extract (Table 1). The AA of water-ethanolic extracts obtained by the DPPH assay was about 4-fold higher on average, compared to water extracts from the investigated plants.

Vallveedú-Queralt et al. [17] reported that the AA of caraway water-ethanolic extract obtained by ABTS was 2.32 mmol TE·g^−1^ and for the DPPH method 1.63 mmol TE·g^−1^. Moreover, this extract was characterised by the strongest AA compared to extracts from turmeric, dill, marjoram and nutmeg. Dobravalskytė et al. [37] confirmed high antioxidant properties of coltsfoot extracts in their investigation. Biel et al. [48] demonstrated that dandelion has a low AA (25.9 µmol TE·100 g^−1^ d.m.), but they used other eluents in their work. However, Ivanov [38] reported that the AA of water-ethanolic extract from dandelion measured by the DPPH method was about 3-fold higher compared to water extract. Sepahpour et al. [49] stated that generally, the antioxidant activity (DPPH) of all the analysed medicinal plant extracts showed the lowest values when water was used for extraction in comparison to organic solvents (methanol, ethanol and aceton). The results presented by Mariutti et al. [50] showed that tarragon exhibits moderate antioxidant properties in comparison to herbs such as rosemary and thyme. However, they also showed that this plant was a stronger antioxidant than caraway, the tendency of which was also noticeable in this study. Mumivand et al. [24] reports that the DPPH activity of tarragon extracts ranged from 0.034 to 0.101 (IC50 mg-mL^−1^) depending on the place of harvest. Memon et al. [44] studied the antioxidant activity of white mulberry extract using the DPPH method and showed that the plant has an average antioxidant activity; however, using 80% methanol as an eluent, the activity of white mulberry extract significantly increased. Sotiropoulou et al. [51] stated that other parameters, like extraction temperature, time and amount of solvent, also play important roles in the evaluation of properties of herbal plants.

Table 3 shows the correlations between the content of caffeic acid derivatives and antioxidant activity in water and water-ethanolic extracts from medicinal plants. It was stated that among the phenolic acids present in water-ethanolic extracts of investigated medicinal plants, the following acids: 3-CQA, 4-CQA, 5-CQA, 3,4-diCQA, 3,5-diCQA and 4,5-diCQA acid, were correlated with antioxidant activity (ABTS and DPPH). Correlation coefficients indicated that correlation in the majority was very high or relatively high.

The highest correlation between phenolic acids and antioxidant activity (ABTS and DPPH) was found for 5-CQA acid (r = 0.995 ** and r = 0.858 **, respectively). Also, high correlation was found for 3,4-diCQA (r = 0.823 ** and r = 0.776 **) respectively, for both assays of antioxidant activity, ABTS and DPPH (Table 3). Regarding the water extracts, the 5-CQA acid content had the highest correlation with ABTS and DPPH (r = 0.623 ** and r = 0.601 *, respectively). Also, 4-CQA acid showed significant correlation for assays of antioxidant activity (ABTS and DPPH), while 3-CQA only for ABTS. However, the 3,4-diCQA (3,4-dicaffeoylquninic acid), 3,5-diCQA (3,5- dicaffeoylquninic acid) and 4,5-diCQA (4.5-dicaffeoylquninic acid) had no significant correlation for antioxidant activity in water extract (Table 3).

Many authors [13,27,52,53] reported that there is a significant correlation between the content of phenolic compounds (especially phenolic acids) in different plants (e.g., medicinal plants, potatoes) and antioxidant activity. Cai et al. [27] found a positive, linear relationship between antioxidant activity (ABTS) and total phenolic content (all R^2^ values ≥ 0.95) in several medicinal herbs.

Alu’datt et al. [53] reported a positive correlation (*r* = 0.69) between the content of phenolic compounds and antioxidant activity in *Rosmarinus officinalis* L., while Zhang et al. [52] reported a positive correlation in methanolic extracts from S. miltiorrhiza. Ru et al. [13] demonstrated that in the free fraction, the content of each individual phenolic acid (in yellow, white, red and purple fleshed potatoes) was positively correlated with antioxidant activity. Cai et al. [27] stated that all analysed medicinal herbs exhibited far stronger antioxidant activity and contained significantly higher levels of phenolics than common vegetables and fruits.

Generally, the application of an appropriate extraction method in plants, including selection of the appropriate eluent to use these extracts in food production, is of great importance, both in terms of obtaining valuable compounds (including phenolic acids) from plants, increasing the antioxidant activity of these compounds and indicating the particular health-promoting plants to food producers and consumers.

## 4. Conclusions

It was found that the plant extracts obtained were characterised by different contents of caffeic acid derivatives, other caffeic acid derivatives and diversified antioxidant activities, which was influenced by both raw material and the type of eluent used. The water-ethanol extracts were characterised on average by about 9-times higher contents of caffeic acids derivatives level than water extract. Both in water and water-ethanolic extracts, the dominant phenolic acid was 5-CQA (5-*O*-caffeoylquinic acid) and 3,4-diCQA (3,4-dicaffeoylquinic acid), then CCA-1 (chicoric acid isomer 1), which appeared only in water-ethanol extract. Extracts from dandelion contained compounds such as CTA (caftaric acid), CCA-1 (chicoric acid isomer 1) and CCA-2 (chicoric acid isomer 2), which were not detected in other plant extracts examined in this work.

The water-ethanolic extract from coltsfoot and tarragon was characterised by a high content of di-caffeoylquinic acids, especially 3,4-diCQA and 3,5-diCQA, respectively. Among the water extracts, the highest content of the total studied compounds was characterised by coltsfoot. None of the caffeic acid derivatives group was found in the water extract from tarragon, although the amounts of the tested acids in the water-ethanolic extract from this plant were significant. The lowest content of total analysed acids was stated in water extract of white mulberry. It was stated that 5-O-caffeoylquinic acid was represented in 40% to 74% of all of the extracted phenolic acids.

Antioxidant activity measured by ABTS and DPPH was higher (on average by almost 1.5-fold and 6-fold, respectively) in extracts obtained with eluent (ethanol) in comparison to water extracts. It was stated that there is a positive correlation between caffeic acid derivatives and antioxidant activity (ABTS and DPPH), especially in water-ethanolic extracts of medicinal plants. The highest correlation between 5-CQA and the antioxidant activity of ABTS and DPPH was found for both water-ethanolic and water extracts. In conclusion, extracts from coltsfoot, dandelion and tarragon appear to be more highly recommended for use in food production and human consumption.

## Figures and Tables

**Table 1:** Content of caffeic acid derivatives in water and water-ethanolic extracts from medicinal plants (mg CQA·g^−1^).

Compound	Medicinal Plant											
	Ca		Col		D		L		T		Wm	
	W	W/E	W	W/E	W	W/E	W	W/E	W	W/E	W	W/E
3-CQA	0.54 ^b^ ± 0.01	0.55 ^A^ ± 0.03	0.78 ^b^ ± 0.02	1.20 ^B^ ± 0.01	n.d.	n.d.	2.15 ^c^ ± 0.06	5.34 ^D^ ± 0.05	n.d.	2.82 ^C^ ± 0.01	0.02 ^a^ ± 0.01	1.58 ^B^ ± 0.03
4-CQA	0.44 ^b^ ± 0.01	0.79 ^A^ ± 0.04	1.59 ^c^ ± 0.03	2.53 ^C^ ± 0.03	n.d.	n.d.	1.01 ^c^ ± 0.02	1.69 ^B^ ± 0.03	n.d.	3.86 ^D^ ± 0.03	0.02 ^a^ ± 0.01	1.97 ^BC^ ± 0.03
5-CQA	1.17 ^b^ ± 0.06	2.23 ^A^ ± 0.09	9.41 ^d^ ± 0.10	20.58 ^D^ ± 0.11	0.52 ^a^ ± 0.02	4.40 ^B^ ± 0.04	4.16 ^c^ ± 0.03	11.66 ^C^ ± 0.10	n.d.	28.92 ^D^ ± 0.11	0.14 ^a^ ± 0.02	7.16 ^B^ ± 0.07
3,4-diCQA	0.13 ^a^ ± 0.03	0.63 ^A^ ± 0.03	3.18 ^b^ ± 0.08	51.58 ^B^ ± 0.10	n.d.	n.d.	n.d.	n.d.	n.d.	1.16 ^A^ ± 0.03	n.d.	n.d.
3,5-diCQA	0.28 ^a^ ± 0.03	1.83 ^A^ ± 0.05	1.62 ^b^ ± 0.06	12.01 ^B^ ± 0.09	n.d.	n.d.	n.d.	n.d.	n.d.	18.58 ^C^ ± 0.08	n.d.	n.d.
4,5-diCQA	0.16 ^a^ ± 0.02	1.40 ^A^ ± 0.04	0.24 ^b^ ± 0.02	13.25 ^C^ ± 0.06	n.d.	n.d.	n.d.	n.d.	n.d.	5.47 ^B^ ± 0.04	n.d.	n.d.
CTA	n.d.	n.d.	n.d.	n.d.	0.39 ± 0.01	11.97 ± 0.08	n.d.	n.d.	n.d.	n.d	n.d.	n.d.
CCA-1	n.d.	n.d.	n.d.	n.d.	n.d.	73.83 ± 0.12	n.d.	n.d.	n.d.	n.d.	n.d.	n.d.
CCA-2	n.d.	n.d.	n.d.	n.d.	n.d.	4.73 ± 0.06	n.d	n.d	n.d.	n.d.	n.d.	n.d.
**TOTAL**	**2.72 ^b^**	**7.43 ^A^**	**16.18 ^d^**	**101.15 ^D^**	**0.91 ^a^**	**94.93 ^D^**	**7.32 ^c^**	**18.69 ^B^**	**n.d**	**60.81 ^C^**	**0.18 ^a^**	**10.71 ^A^**

Ca—caraway; Col—Coltsfoot; D-dandelion; L—lovage; T—tarragon; Wm—white mulberry; W—water extracts; W/E—water-ethanolic extracts; 3-CQA—(3-*O*-caffeoylquinic acid; neochlorogenic acid); 4-CQA—(4-*O*-caffeoylquinic acid; cryptochlorogenic acid); 5-CQA—(5-*O*-caffeoylquinic acid; chlorogenic acid); 3,4-diCQA—3,4-dicaffeoylquinic acid; 3,5-diCQA—3,5-dicaffeoylquinic acid; 4,5-diCQA—4,5-dicaffeoylquinic acid.; CTA—caftaric acid; CCA-1—chicoric acid isomer 1; CCA-2—chicoric acid isomer 2; n.d.—non detected; d.w.—dry weight) (±SD)—standard deviation (*n* = 9); Values in each row (W) with different letters (a–d) are significantly different (*p* ˂ 0.05). Values in each row (W/E) with different capital letters (A–D) are significantly different (*p* ˂ 0.05).

**Table 2:** Antioxidant activity determined with ABTS (µmol TE·g^−1^ d.m.) and DPPH (µmol TE·g^−1^ d.m.) assay in water and water-ethanolic extracts from medicinal plants.

Antioxidant Activity	Medicinal Plant											
	Ca		Col		D		L		T		Wm	
	W	W/E	W	W/E	W	W/E	W	W/E	W	W/E	W	W/E
ABTS	144.06 ^b^ ± 0.17	182.84 ^A^ ± 0.86	320.80 ^d^ ± 1.09	390.00 ^D^ ± 1.13	165.45 ^b^ ± 1.11	277.25 ^B^ ± 1.02	194.44 ^c^ ± 0.88	331.03 ^C^ ± 0.99	224.13 ^c^ ± 0.26	406.29 ^D^ ± 1.14	59.42 ^a^ ± 0.08	180.74 ^A^ ± 0.98
DPPH	31.65 ^b^ ± 0.10	107.52 ^B^ ± 0.81	51.63 ^d^ ± 0.09	268.52 ^D^ ± 1.06	31.56 ^b^ ± 0.08	95.06 ^A^ ± 0.98	49.24 ^cd^ ± 0.07	182.19 ^C^ ± 0.32	42.99 ^c^ ± 0.07	117.51 ^B^ ± 1.01	18.83 ^a^ ± 0.05	87.77 ^A^ ± 0.07

Ca-caraway; Col—coltsfoot; D—dandelion; L—lovage; T—tarragon; Wm—white mulberry; W—water extracts; W/E—water-ethanolic extracts; n.d.—not detected; d.m.—dry matter; (±SD)—standard deviation (*n* = 9); Values in each row (W) with different letters (a–d) are significantly different (*p* ˂ 0.05). Values in each row (W/E) with different capital letters (A–D) are significantly different (*p* ˂ 0.05).

**Table 3:** Correlation coefficients (*r*) between different caffeic acid derivatives and antioxidant activity in water and water-ethanolic extracts from medicinal plants.

Caffeic Acid Derivatives		Water-Ethanolic Extracts		Water Extracts
	ABTS	DPPH	ABTS	DPPH
3-CQA	0.653 **	0.593 *	0.503 *	n.s
4-CQA	0.798 **	0.715 **	0.542 *	0.502 *
5-CQA	0.995 **	0.858 **	0.623 **	0.601 *
3,4-diCQA	0.823 **	0.776 **	n.s	n.s
3,5-diCQA	0.603 *	0.759 **	n.s	n.s
4,5-diCQA	0.673 **	0.574 *	n.s	n.s

W—water extracts; W/E—water-ethanolic extracts; 3-CQA—(3-*O*-caffeoylquinic acid; neochlorogenic acid); 4-CQA—(4-*O*-caffeoylquinic acid; cryptochlorogenic acid); 5-CQA—(5-*O*-caffeoylquinic acid; chlorogenic acid); 3,4-diCQA—3,4-dicaffeoylquinic acid; 3,5-diCQA—3,5-dicaffeoylquinic acid; 4,5-diCQA—4,5-dicaffeoylquinic acid.; n.s.—no significant differences; * and ** indicate significance at (*p* ˂ 0.05 and *p* ˂ 0.01).
